# Genetic Basis of High-Pressure Tolerance of a *Vibrio parahaemolyticus* Mutant and Its Pathogenicity

**DOI:** 10.3389/fmicb.2022.827856

**Published:** 2022-03-31

**Authors:** Lifang Feng, Minhui Xu, Junli Zhu, Haixia Lu

**Affiliations:** College of Food Science and Biotechnology, Zhejiang Gongshang University, Hangzhou, China

**Keywords:** *Vibrio parahaemolyticus*, high-pressure processing, adaptive laboratory evolution, comparative genomic analysis, *mlaC* and *mlaD*, tolerance, pathogenicity

## Abstract

Foodborne pathogens with high-pressure processing (HPP) tolerance and their pathogenicity have gained considerable attention in the field of food safety. However, tolerance to pressure treatment varies among microorganisms and growth phases, and the mechanism by which *Vibrio parahaemolyticus* can become tolerant of HPP is currently not known. In this study, 183 strains of *V. parahaemolyticus* were isolated from seafood products, and one strain, C4, carried a thermostable direct hemolysin (*tdh*) gene. A strain, N11, which was acquired from the C4 strain through adaptive laboratory evolution under HPP stress, could tolerate up to 200 MPa for 10 min. Compared with the C4 strain, the catalase and Na^+^/K^+^-ATPase activities in N11 strain were increased by about 2–3 times, and the cells maintained an intact cell membrane structure under HPP treatment. As shown by murine infection trials, the C4 and N11 strains impacted the physiological activities of mice and damaged liver and spleen cells. Comparative genomic analysis showed that 19 nucleotides were mutated in the N11 strain, which led to sustained high expression of *mlaC* and *mlaD* genes in this strain. Knockout of these genes confirmed that they were involved in the high-pressure stress response, and also related to pathogenicity of *V. parahaemolyticus*. Thereby, our findings revealed a HPP tolerance mechanism of *V. parahaemolyticus*, and the high-pressure-tolerant strain still retained pathogenicity in mice with skin and fur pleating and lethargy, indicating the pressure-tolerant foodborne pathogens present health risks.

## Introduction

As a Gram-negative halophilic foodborne pathogen, *Vibrio parahaemolyticus* is present in seawater, seabed sediment, and seafood products, and it may cause gastroenteritis, diarrhea, and fever in people following the consumption of raw or undercooked seafood (Raszl et al., 2016). Although *V. parahaemolyticus* has a high detection rate in seafood, not all of its strains are pathogenic, and its pathogenicity mainly depends on virulence factors (Yang et al., 2017; Li et al., 2019). The main two virulence genes are thermostable direct hemolysin (*tdh*) and TDH-related hemolysin (*trh*) (Raghunath, 2014). The type III secretion system (T3SS) is another virulence factor, with T3SS1 and T3SS2 contributing to cytotoxicity and enterotoxicity, respectively (Lovell, 2017; Miller et al., 2019). Another secretory device is the type IV secretion system (T6SS), which secretes effectors into host cells; both T6SS1 and T6SS2 can be present in clinical and environmental isolates. T6SS2 performs the first step of infection, while T3SS2 exports effector proteins directly into the host cell cytoplasm (Ben-Yaakov and Salomon, 2019). Initial host cell binding is the most important step in pathogenesis. Multivalent adhesion molecule 7 (MAM7) is an adhesin that is widely distributed in Gram-negative pathogens. MAM7 contains seven mammalian cell entry domains that help bacteria bind to the host protein fibronectin and membrane phosphatidic acid (Liu et al., 2018).

High-pressure processing (HPP) is used to inactive microorganisms in food without losing nutrients and enables the use of pressures much higher than those in traditional homogenizers; the pressure range in most commercial applications is 100–600 MPa, with some employing pressures up to 1,000 MPa (Demazeau and Rivalain, 2011; Santos et al., 2017). HPP has been applied as a physical process in food and equipment enterprises because of its advantages of providing rapid, quasi-instantaneous distribution throughout the sample; being suitable for high moisture-content, liquid, and pumpable foods. HPP can produce protein denaturation, carbohydrate gelatinization, and fat crystallization (Marangoni Júnior et al., 2019). The United States Food and Drug Administration approved the application of HPP for commercial sterilization of preheated low-acid foods in 2009 (Balasubramaniam et al., 2015). Since then, HPP has been increasingly used in the production of a wide range of food products, such as meat and seafood products, fruits, and vegetables, due to the technological development of high-pressure vessels withstanding up to 1000s of pressure cycles (Wang et al., 2016; Argyri et al., 2018).

The mechanism of high-pressure-induced inactivation of microorganisms involves cell membrane lipid damage, intracellular pH shifts by solute transport, metal ion release, enzyme deactivation, and decreased DNA synthesis (Patrignani and Lanciotti, 2016; Nikparvar et al., 2021). Unfortunately, a small number of both Gram-positive and Gram-negative bacteria can survive after 400–600 MPa HPP treatment for 8 min (Li et al., 2020), and their survival might lead to contamination with living microbes that can pose a threat to human health.

In this study, a pathogenic strain of *V. parahaemolyticus* containing thermolabile hemolysin (*tlh*) and *tdh* genes was isolated from an oyster. After eight rounds of HPP treatment, this strain evolved into a strain that could resist 200 MPa treatment for 10 min. The physiological characteristics and pathogenicity of this high-pressure-tolerant strain were investigated, and a high-pressure tolerance mechanism was revealed by comparative genome analysis and gene knockout (KO) methods.

## Materials and Methods

### Strain Isolation and Identification

A total of 152 samples of oyster (63), clam (42), scallop (31), and large yellow croaker (16) were purchased from Jinjiang Aquatic Market (Hangzhou City, China) from June 3, 2014 to October 28, 2014. Each seafood sample was individually packed in a plastic bag and transported on bagged ice to the laboratory within one hour. The shellfish samples were scrubbed and shucked. A sample (25 g) of fish or shell contents was collected aseptically in a vertical laminar-flow cabinet and transferred into a sterile stomacher bag containing 225 mL of alkaline peptone water (APW) supplemented with 3% NaCl. The mixture was homogenized for 2 min using a Stomacher 400 (Seward, Worthing, West Sussex, United Kingdom).

Serial decimal dilutions were made with APW supplemented with 3% NaCl and streaked on a thiosulfate-citrate-bile salt-sucrose (TCBS) agar plate and incubated at 37°C for 18–24 h. A single colony was picked and cultured in APW supplemented with 3% NaCl and subsequently tested for its morphological and biochemical characteristics by Gram staining, oxidase reaction, evaluation of salt concentration effects on growth, and glucose and lactose fermentation. Based on the morphological and biochemical results, the contamination of *V. parahaemolyticus* in seafood samples was quantified by most probable number (MPN) technique according to the positive and negative tubes at each dilution within the estimated 95% confidence interval (Williams and Ebel, 2012).

After morphological and biochemical testing, all the isolates were cultured in APW supplemented with 3% NaCl overnight and then spun down by centrifugation to obtain pellets. The genomic DNA of each isolate was extracted using a MiniBEST Bacterial Genomic DNA Extraction Kit (TaKaRa, Dalian, China) and subsequently amplified by PCR using primers for *tlh*, *tdh*, and *trh* (Supplementary Table 1). According to the results of PCR amplification with the *tdh* primer, only one strain, C4, contained a *tdh* gene.

Genomic DNA of the C4 strain was used as template and amplified with 16S and toxR primers (Supplementary Table 1). The PCR products were cloned into pGEM-T (Promega, Madison, WI, United States) and transformed into *Escherichia coli* DH5α. White colonies were selected, and their plasmids were extracted and sequenced with M13 primers (Supplementary Table 1). The sequences of 16S rRNA were aligned by MEGA software (version 7.0), and a phylogenetic tree was constructed by the neighbor-joining method with 1,000 bootstrap replicates. The identity values of the *toxR* gene sequences of different *Vibrio* strains were calculated with DNASTAR software. All primer synthesis and DNA sequencing were performed by Sangon Biotech (Shanghai) Co., Ltd., China. All culture media were purchased from Qingdao Hope Bio-Technology Co., Ltd., Qingdao, China.

### Screening of the High-Pressure-Tolerant Strain

The C4 strain was cultured in APW supplemented with 3% NaCl and collected by centrifugation until the cells reached the stationary phase. After washing twice in sterile saline water, the cell density was adjusted with sterile saline water to 10^8^CFU/mL, and cells were transferred to a sterile sampling bag that was sealed without any headspace. High-pressure treatment was administered using a high-pressure vessel (HPB. A2-600/0.6, Tianjin Huatai Senmiao Bioengineering Technology Inc. Co., Tianjin, China) with water as the transmission fluid. There were eight rounds of high-pressure treatment, applied in sequence from low to high: 80, 100, 120, 150, 180, 200, 200, and 200 MPa. The treatment time of each round was 10 min. After each round of high-pressure treatment, the bacteria were cultured on tryptic soy agar (TSA) plates with 3% NaCl, and a single colony was picked to enter the next round of high-pressure treatment. At the end of the high-pressure treatment, the surviving strain was tolerant to a pressure of 200 MPa for 10 min and was named N11.

### Evaluation of Physiological Characteristics

To produce a growth curve, the C4 and N11 strains were cultured in APW supplemented with 3% NaCl with shaking at 150 rpm at 37°C. The cell density was measured according to the *OD*_600_ values by an Ultrospec 2100 UN/Visible spectrophotometer (Golden Bio Technologies Corporation, Jersey City, NJ, United States).

To compare HPP tolerance between the C4 and N11 strains, both strains were subjected to one of several HPP treatments for 10 min: 100, 150, 200, 250, and 300 MPa. Subsequently, 1 mL of treated cells was serially diluted and cultured on plate count agar (PCA) plates. After incubation at 37°C for 24 h, the total number of colony forming units (CFUs) was considered the total viable count (TVC).

Cells of C4 and N11 were treated with HPP for 10 min at 100, 150, 200, or 250 MPa. The activities of Na^+^/K^+^-ATPase, lactate dehydrogenase (LDH), and catalase (CAT) were determined by enzymatic assay kits (Nanjing Jiancheng Bioengineering Institute, Nanjing, China), and the zeta potential was measured by a Zetasizer Nano (Malvern, Worcestershire, United Kingdom) as described previously (Feng et al., 2018).

### Examination of the Cell Membrane by Transmission Electron Microscopy

C4 and N11 cells were treated with a high pressure of 200 MPa for 10 min; untreated cells were used as controls. The next steps of cell fixation, dehydration, embedding, and transmission electron microscopy (TEM) examination were performed as described previously (Feng et al., 2018).

### *In vitro* Virulence Test

To test the virulence of the C4 and N11 strains *in vitro*, cells were spread onto the surface of Wagatsuma agar containing 5% defibrillated sheep whole blood and 7% NaCl and incubated at 37°C for 14 h. Biofilm formation was measured using the method of Syal (2017). The two strains were cultured in APW medium supplemented with 3% NaCl at 37°C, and their biofilms were stained with 0.1% (w/v) crystal violet. Then the biofilm was quantified by measuring the absorbance at 595 nm (*OD*_595_).

### *In vivo* Virulence Test

An acute toxicity test in mice was used to evaluate the toxicity of the C4 and N11 strains *in vivo*. Forty eight female mice of the ICR strain, 5 weeks old and weighing 18 ± 2 g, were randomly divided into eight cages, with six mice per cage. The mice were provided sterile food and water *ad libitum* and housed in cages for 1 week prior to dosing to allow for acclimatization to the laboratory conditions. Before bacterial challenge for 24 h, food was withdrawn, but water was still given *ad libitum*. Cells of C4 and N11 were prepared at densities of 10^8^, 10^7^, and 10^6^CFU/mL in phosphate-buffered saline (PBS) and administered at a dose of 0.1 mL per mouse by oral gavage. The treated mice had free access to food and sterile water after inoculation. After 24 h, changes in skin, fur, somatomotor activity, behavior pattern, and diarrhea were observed. Then, the mice were euthanized by ether inhalation, and blood samples were collected. The four components of white blood cells (WBCs), red blood cells (RBCs), hemoglobin (HGB), and platelets (PLTs) were evaluated by a Cell-Dyn 3700 Hematology Analyzer (Abbott Park, IL, United States). Furthermore, serum was separated from blood by centrifugation, and aspartate aminotransferase (AST), alanine aminotransferase (ALT), alkaline phosphatase (ALP), and blood urea nitrogen (BUN) levels were measured by enzymatic assay kits (Nanjing Jiancheng Bioengineering Institute, Nanjing, China). The animal study was reviewed and approved by the School Animal Care and Ethics Committee of Zhejiang Gongshang University.

### Histological Examination

After the *in vivo* virulence test, the liver and spleen of the mice were immediately acquired aseptically and fixed in 10% (v/v) neutral buffered formalin for 24 h. The tissues were dehydrated, mounted, and stained with hematoxylin and eosin as described by Jaiswal et al. (2014).

### Genome Sequencing and Comparative Genome Analysis

The genomic DNA of the N11 and C4 strains was prepared and qualified by agarose gel electrophoresis and a bioanalyzer (2100, Agilent Technologies, Santa Clara, CA, United States). The DNA was submitted to Genergy Bio-Technology (Shanghai, China) Co., Ltd. Mate-pair (MP) and paired-end (PE) libraries were constructed for the C4 strain, and a PE library was constructed for the N11 strain. Genomic sequencing was performed on the Illumina HiSeq 2000 platform. Genome assembly, gene prediction, and gene functional annotation of the C4 and N11 strains were performed as described previously (Zhu et al., 2019). Variant discovery between the C4 and N11 strains and variant genotyping were performed with GATK software, and SNPs and indels were identified with BCFtools software (Narasimhan et al., 2016). Furthermore, fragments containing mutated nucleotides were amplified by routine PCR and subjected to Sanger sequencing.

### Determination of Gene Expression Levels

To analyze gene expression levels, real-time quantitative PCR (qPCR) was carried out with the QuantStudio 6 Flex Real-Time PCR System (Applied Biosystems, Carlsbad, CA, United States). After treatment with HPP for 10 min at 100, 150, 200, or 250 MPa, C4 and N11 cells were collected. Their total RNA was extracted by using TRIzol reagent (Invitrogen, Carlsbad, CA, United States), digested by DNase (Promega, Madison, WI, United States) and reverse transcribed into cDNA. The 16S rRNA gene was employed as a housekeeping gene (Supplementary Table 1). Expression levels were calculated as described previously (Feng et al., 2018).

### Construction and Virulence Testing of Knockout Strains

The upstream and downstream sequences of the target gene were amplified from the genomic DNA of the C4 strain, and the cassette of the chloramphenicol (Cm) acetyltransferase gene was amplified from the genomic DNA of the pKD3 plasmid. These three PCR fragments were joined using nested PCR. Transformation, testing, and screening were performed as described previously (Zhu et al., 2019). Virulence tests of the KO strains were performed as described in section “*In vivo* Virulence Test.”

### Statistical Analyses

All analyses were carried out in triplicate, and the results are reported as the mean values and standard deviations. One-way analysis of variance (ANOVA) was performed for all data, and mean separation was performed by Tukey’s multiple range test implemented with SPSS software. Differences were considered significant at *p*-value < 0.05.

### Omics Data

All Illumina DNA sequencing data were submitted to the NCBI Short Read Archive^1^ with the accession number PRJNA645751.

## Results

### Analysis of Contamination Status and Identification of *Vibrio parahaemolyticus* in Four Kinds of Seafood

**FIGURE 1:**
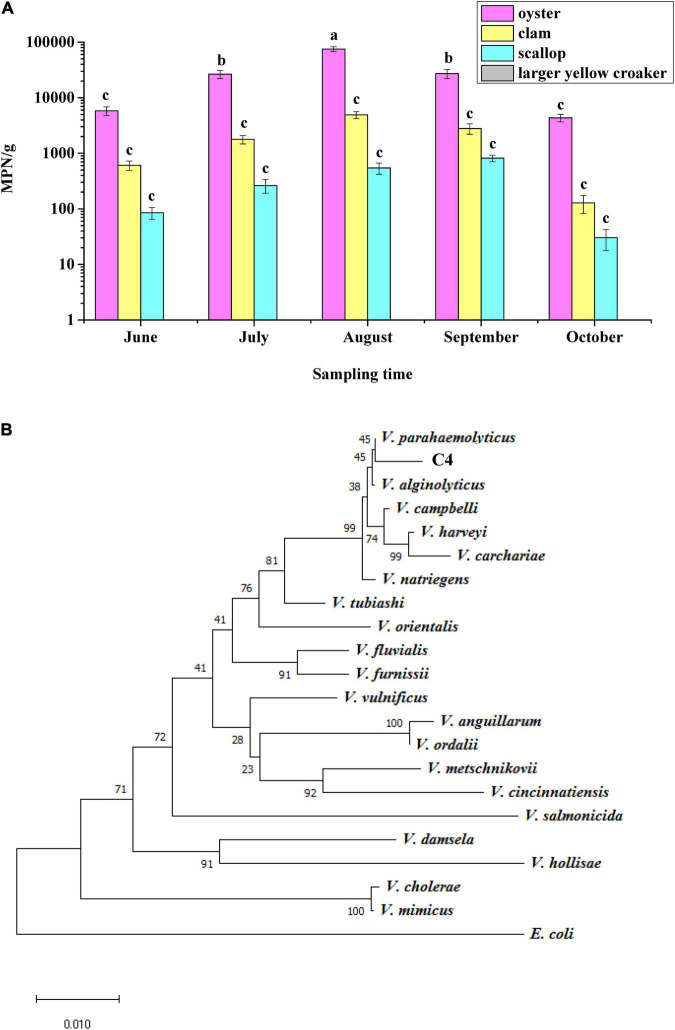
Identification of *Vibrio parahaemolyticus*. **(A)** Analysis of the status of *V. parahaemolyticus* contamination in four kinds of seafood. **(B)** Phylogenetic tree based on 16S rRNA gene sequences showing that the C4 strain represents a subgroup within the genus *V. parahaemolyticus*.

In this study, 152 samples of oyster, clam, scallop, and large yellow croaker were purchased in Hangzhou City in summer, and 183 strains of *V. parahaemolyticus* were identified according to their morphological and biochemical characteristics (Supplementary Table 2). Samples of oyster, clam, and scallop were contaminated with *V. parahaemolyticus*, among which oyster had the highest count, 7.5 × 10^4^ MPN/g, in August, but it was not detected in the large yellow croaker samples (Figure 1A), indicating that different types of seafood carry different amounts of *V. parahaemolyticus*. Moreover, July, August, and September were the months with the highest levels of *V. parahaemolyticus* contamination.

To verify the results regarding morphological and biochemical characteristics, all isolates were amplified with the *tlh* primer, and 185 strains were shown to be positive for this gene, including the above 183 strains. When primers for *tdh* and *trh* were used to test these 185 strains, it was found that only one strain contained the *tdh* gene; this strain was named C4 and considered as a wild-type (WT) strain. The *trh* gene was not detected in all strains. In addition, a phylogenetic tree was constructed based on 16S rRNA gene sequences, and the results showed that the C4 strain shared high sequence similarity with the corresponding *V. parahaemolyticus* gene (Figure 1B). Furthermore, the *toxR* gene sequences of eight *Vibrio* strains were aligned, and the C4 strain shared 99% nucleotide identity with that of *V. parahaemolyticus* (Supplementary Table 3). Therefore, the C4 strain was identified as *V. parahaemolyticus*.

### Physiological Characteristic of High-Pressure-Tolerant Strain Acquired by Adaptive Laboratory Evolution

**FIGURE 2:**
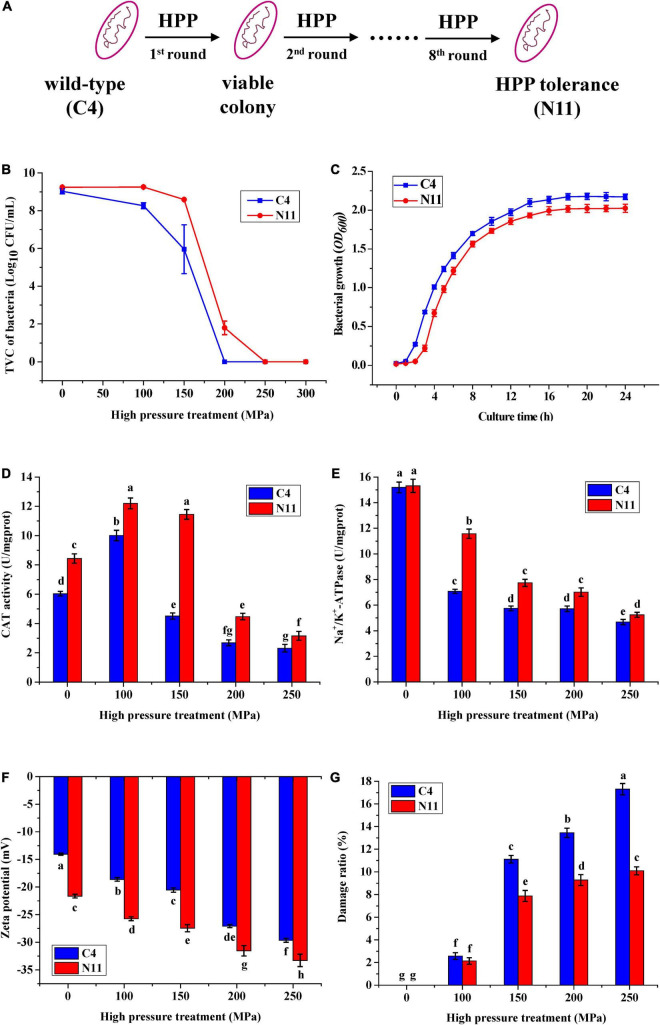
Differences in physiological characteristics between the wild-type (WT) strain and the high-pressure-tolerant strain. **(A)** A schematic of screening of high-pressure processing (HPP)-tolerant strains using adaptive laboratory evolution. **(B)** High pressure tolerance of the C4 and N11 strains under high-pressure treatment. **(C)** Growth curve. **(D)** Catalase (CAT) activity. **(E)** Na^+^/K^+^-ATPase activity. **(F)** Zeta potential. **(G)** Cell membrane permeability was assessed by observing the lactate dehydrogenase (LDH) content.

Under a high pressure of 200 MPa for 10 min, the WT strain of C4 was completely killed. However, after eight rounds of high-pressure treatment from 80 to 200 MPa, the C4 strain evolved into a strain (named N11) that could tolerate 200 MPa treatment (Figures 2A,B), and strains C4 and N11 had similar growth curves (Figure 2C).

Under 100 MPa pressure treatment, the catalase (CAT) activities of the C4 strain and N11 strain were increased by 42 and 67%, respectively, compared with their non-HPP treatment groups (Figure 2D). With increasing HPP stress (100–250 MPa), the activities of CAT in these two strains gradually decreased, which might have been due to the destruction of the enzymatic structure by HPP. In addition, the CAT activity of the N11 strain was consistently higher than that of the C4 strain, differing by nearly threefold under 150 MPa treatment, suggesting that CAT in *V. parahaemolyticus* conferred protection to bacteria under high-pressure treatment and might be an important factor enabling the N11 strain to resist high pressure.

High-pressure stress effectively inhibited the activity of Na^+^/K^+^-ATPase in the C4 strain (Figure 2E). Under 100 MPa treatment, the activity of Na^+^/K^+^-ATPase in the C4 strain was only half of the non-HPP treatment groups. However, the decrease in the N11 strain was smaller, with 76.5% Na^+^/K^+^-ATPase activity persisting under the same high-pressure treatment, indicating that Na^+^/K^+^-ATPase was another important factor in enabling the N11 strain to withstand high pressure.

With increasing pressure, the zeta potentials of the C4 strain and N11 strain decreased gradually (Figure 2F). In addition, under the same treatment, the zeta potential of the N11 strain was consistently lower than that of the C4 strain, suggesting that the polarity of the bacterial surface was affected and the integrity of the cell membrane was enhanced in the high-pressure-tolerant strain of N11.

Almost all living cells contain LDH, which catalyzes the conversion between lactate and pyruvate. As a soluble cytoplasmic enzyme, LDH can be released into the extracellular space if the plasma membrane is damaged (Parhamifar et al., 2019). As the pressure increased, the proportions of damaged C4 and N11 cells gradually increased (Figure 2G). Moreover, under the same treatment, the degree of cell damage was higher in the C4 strain than that of the N11 strain.

**FIGURE 3:**
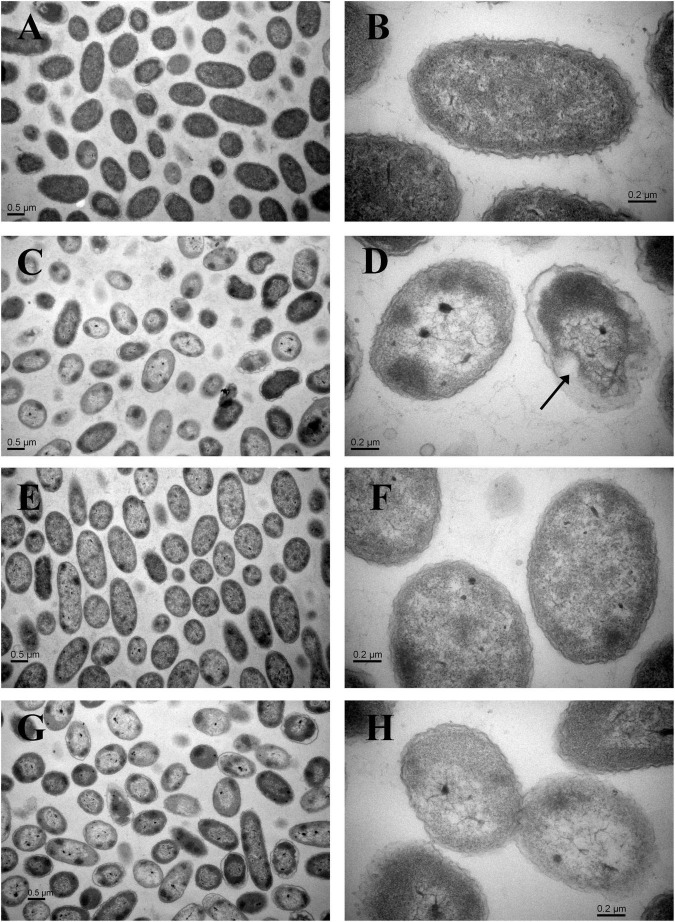
Transmission electron microscopy (TEM) examination of cells. **(A)** Wide view of the C4 strain without HPP treatment. **(B)** Magnified view of the C4 strain without HPP treatment. **(C)** Wide view of the C4 strain following 200 MPa HPP treatment for 10 min. **(D)** Magnified view of the C4 strain following 200 MPa HPP treatment for 10 min. **(E)** Wide view of the N11 strain without HPP treatment. **(F)** Magnified view of the N11 strain without HPP treatment. **(G)** Wide view of the N11 strain following 200 MPa HPP treatment for 10 min. **(H)** Magnified view of the N11 strain following 200 MPa HPP treatment for 10 min.

Compared with the non-HPP treatments (Figures 3A,B,E,F), the cells of the C4 strain showed irregular shapes and rupture after high-pressure treatment (Figures 3C,D), which resulted in the loss of cell contents (e.g., LDH) and cell death. The N11 strain maintained an intact cell membrane and uniform cell cytoplasm under the same treatment (Figures 3G,H), suggesting that the N11 strain had a stronger cell membrane that protected the cells from high-pressure stress.

### Pathogenicity Differences Between the WT Strain and the High-Pressure-Tolerant Strain

**FIGURE 4:**
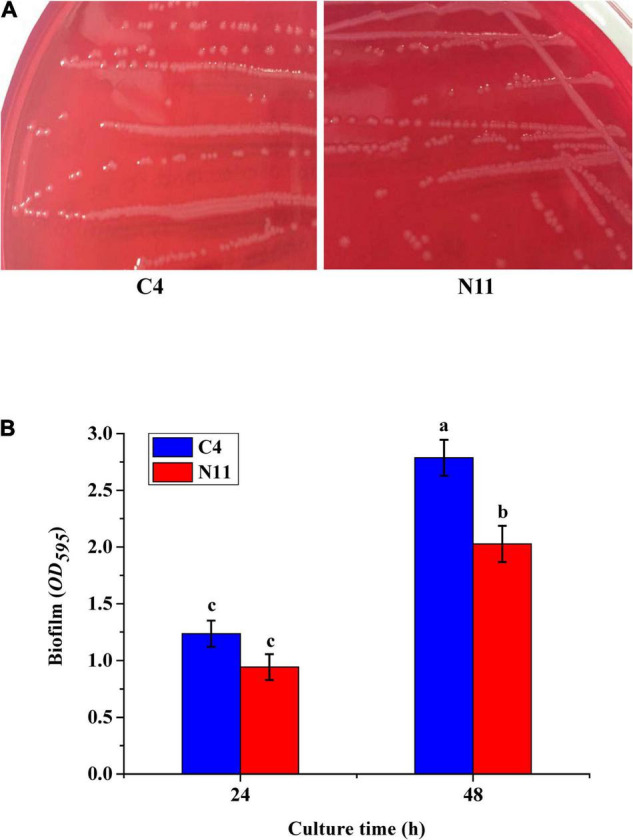
*In vitro* virulence test results of the C4 and N11 strains. **(A)** Kanagawa phenomenon. **(B)** Biofilms formed by different strains after culture for 24 and 48 h.

Cells of *V. parahaemolyticus* producing TDH can lyse RBCs on Wagatsuma agar, which is known as the Kanagawa phenomenon (Cai and Zhang, 2018). In this study, the *tdh* gene was detected only in the C4 strain, which showed the typical Kanagawa phenomenon: a transparent clear zone of blood cells was found around the colony (Figure 4A). The N11 strain, evolved from the C4 strain, yielded a similar Kanagawa phenomenon. Beshiru and Igbinosa (2018) revealed a correlation between biofilm formation and virulence in *Vibrio*. Figure 4B shows that the biofilm amount of the N11 strain was lower than that of the C4 strain, which suggests that the pathogenicity of the N11 strain might be lower than that of the C4 strain.

Murine infection with *V. parahaemolyticus* by oral gavage was conducted to verify a possibility that the N11 strain retained pathogenicity. Based on the changes in skin, fur, somatomotor activity, behavior pattern, lethargy, tremors, and diarrhea (Table 1), the C4 strain and N11 strain were identified as pathogenic, with the pathogenicity of the N11 strain being slightly weaker than that of the C4 strain at the same bacterial density.

**TABLE 1:** Observations of mice after oral gavage with *Vibrio parahaemolyticus*.

Group	Cell density (CFU/mL)	Observation	
		Treatment of C4 strain	Treatment of N11 strain
High	1 × 10^8^	Diarrhea, tremors	Skin and fur pleating, lethargy
Medium	1 × 10^7^	Skin and fur pleating, lethargy	Reduced somatomotor activity, lack of movement
Low	1 × 10^6^	Reduced somatomotor activity, lack of movement	Active and responsive to experimenter intervention
Control	0	Active and responsive to experimenter intervention	Active and responsive to experimenter intervention

**FIGURE 5:**
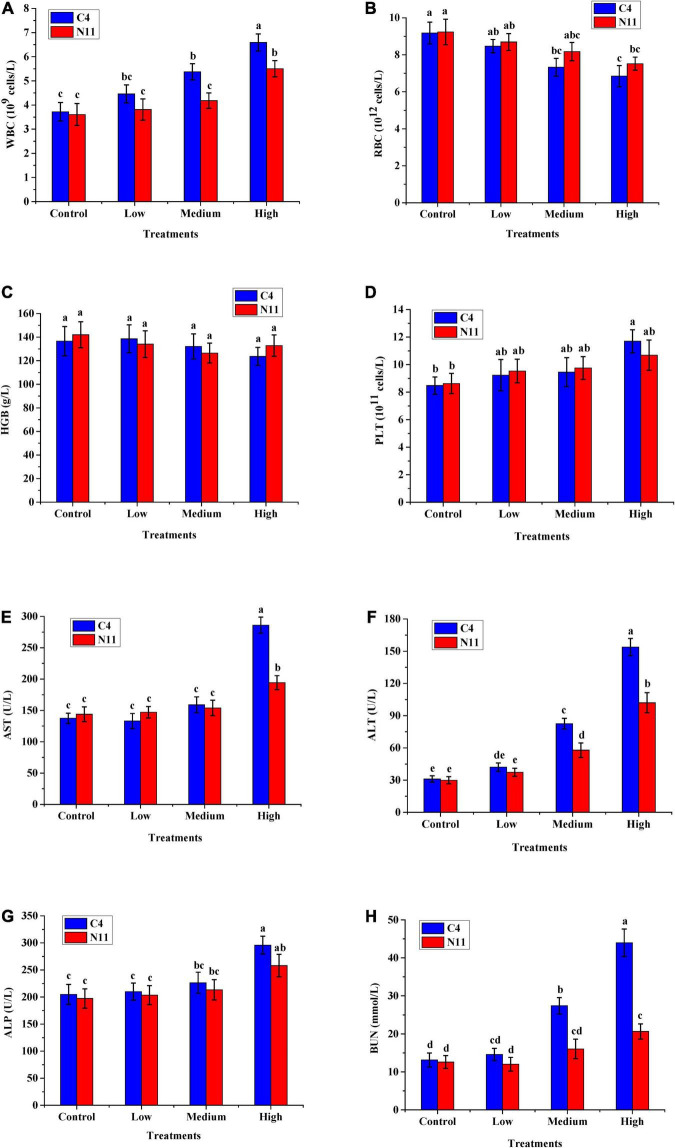
*In vivo* virulence test results of the C4 and N11 strains 24 h after infection of mice by oral gavage. **(A)** White blood cells (WBC); **(B)** Red blood cells (RBC); **(C)** Hemoglobin (HGB); **(D)** Platelets (PLT); **(E)** Aminotransferase (AST); **(F)** Alanine aminotransferase (ALT); **(G)** Alkaline phosphatase (ALP); **(H)** Blood urea nitrogen (BUN).

Four hematological indicators, WBC, RBC, HGB, and PLT, were tested, and the level of WBC was found to increase with increasing bacterial density of *V. parahaemolyticus* in mice. The other four indicators, AST, ALT, ALP, and BUN, which are associated with liver and kidney health, all increased with increasing bacterial density (Figure 5). In addition, under medium- or high-density bacterial exposure, the levels of WBC, AST, ALT, ALP, and BUN in mice infected with the C4 strain were much higher than those in mice infected with the N11 strain, indicating that the pathogenicity of the C4 strain was stronger than that of the N11 strain.

**FIGURE 6:**
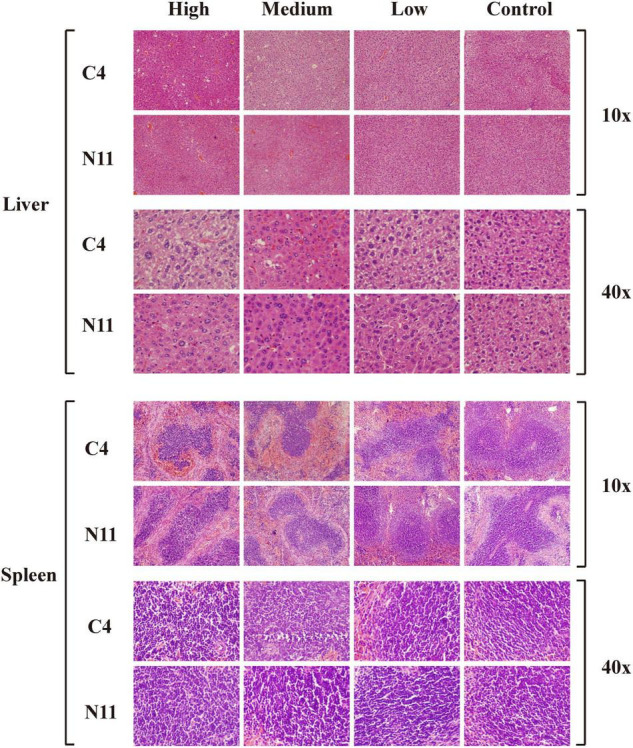
Histological assessment of the liver and spleen of mice after infection by C4 and N11 strains.

Furthermore, histological assessment of liver and spleen after *V. parahaemolyticus* infection was performed (Figure 6). In the high-density infection group of mice infected with the C4 strain, the liver cells were loose, the cell membranes were ruptured, the cytoplasm exuded red liquid, and the cells exhibited vacuoles of different sizes. In contrast, in the control group, the liver cells were arranged in an orderly fashion and evenly colored, with clear structure and normal morphology. These results showed that as the density of bacteria increased, the liver suffered more damage. Under the same high-density bacterial exposure, the liver damage of mice infected with the C4 strain was slightly more severe than that of the N11 strain. The histological results of mouse spleen yielded a similar conclusion to that drawn for the liver; that is, both the C4 strain and N11 strain could damage the mouse spleen, with the C4 strain being slightly more harmful.

### Genomic Analysis of Differences Between the WT Strain and the High-Pressure-Tolerant Strain

To reveal the molecular mechanism of HPP tolerance in *V. parahaemolyticus*, the whole genomes of both the C4 and N11 strains were sequenced. A total of 12,342,947 and 10,309,208 reads were obtained by sequencing the PE and MP libraries, respectively, of the C4 strain. Twelve scaffolds were assembled, and the genome size of the C4 strain was found to be 5,271,691 bp, with a genome GC content of 45.3% (Supplementary Table 4). The ORF number was 4,850, and the non-coding RNA included 110 tRNA and 11 rRNA operons (Supplementary Table 5 and Supplementary Figure 1).

**FIGURE 7:**
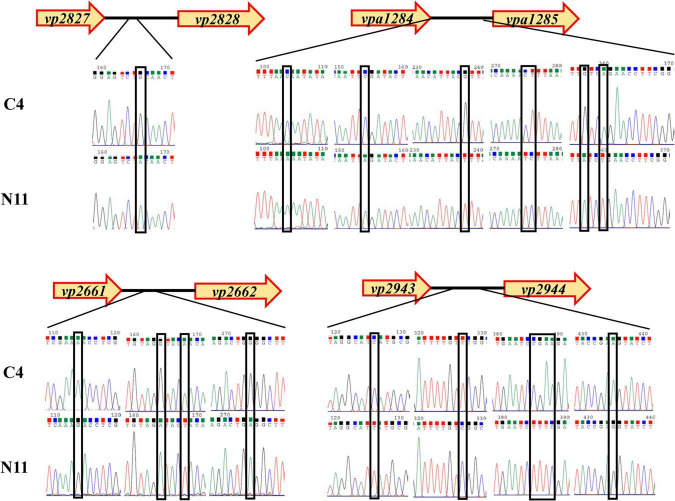
Analysis of mutated nucleotides between the C4 strain and N11 strain by Sanger sequencing.

After sequencing the whole genome of the N11 strain and comparing it with that of the C4 strain, 19 mutated nucleotides were screened from the N11 strain (Supplementary Table 6) and were verified by Sanger sequencing (Figure 7). Surprisingly, all these mutated nucleotides were distributed in the intergenic regions between *vp2661* and *vp2662*, *vp2827* and *vp2828*, *vp2943*, and *vp2944*, and *vpa1284* and *vpa1285*, and those numbered gene names were consistent with those in the RIMD 2210633 strain (Makino et al., 2003). This raises the question of whether these mutated nucleotides affect the expression of neighboring genes.

**FIGURE 8:**
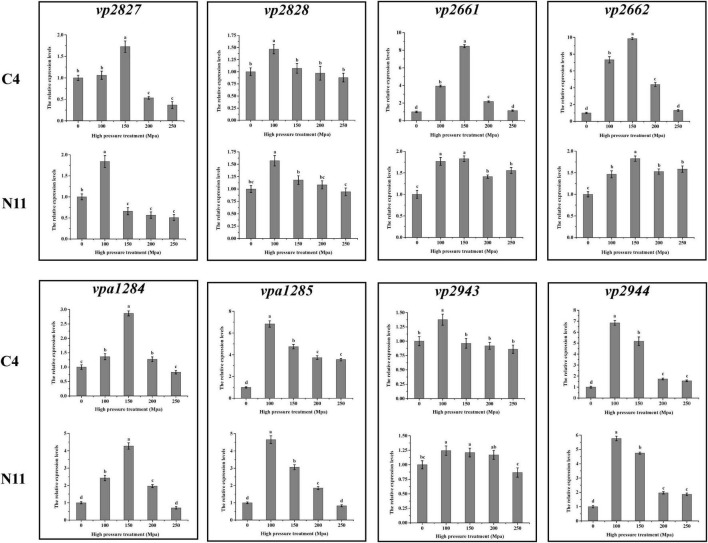
Gene expression levels determined by qPCR.

To seek the answer to this question, the expression levels of the above eight genes under high-pressure treatment were determined by qPCR (Figure 8), and the genes were divided into three groups. The genes in the first group (*vp2661* and *vp2662*) were highly expressed in the C4 strain but not in the N11 strain under the 100 MPa and 150 MPa treatments. The *vp2661* and *vp2662* genes were annotated as the intermembrane phospholipid-binding protein MlaC and the outer membrane lipid asymmetry maintenance protein MlaD, respectively. Mutated nucleotides might result in the constant high expression of *vp2661* and *vp2662* in the N11 strain, thereby conferring the strain tolerance to high pressure. The genes in the second group (*vp2944*, *vpa1284, and vpa1285*) were highly expressed in both the C4 and N11 strains under the 100 and 150 MPa treatments, and their expression levels were not affected by mutated nucleotides. Expression in the third group of genes (*vp2827*, *vp2828*, and *vp2943*) did not change significantly under high pressure. Therefore, the first group of genes (*mlaC* and *mlaD*) might play important roles in the tolerance of *V. parahaemolyticus* to high pressure.

### The *mlaC* and *mlaD* Genes Confer High-Pressure Tolerance in *Vibrio parahaemolyticus*

**FIGURE 9:**
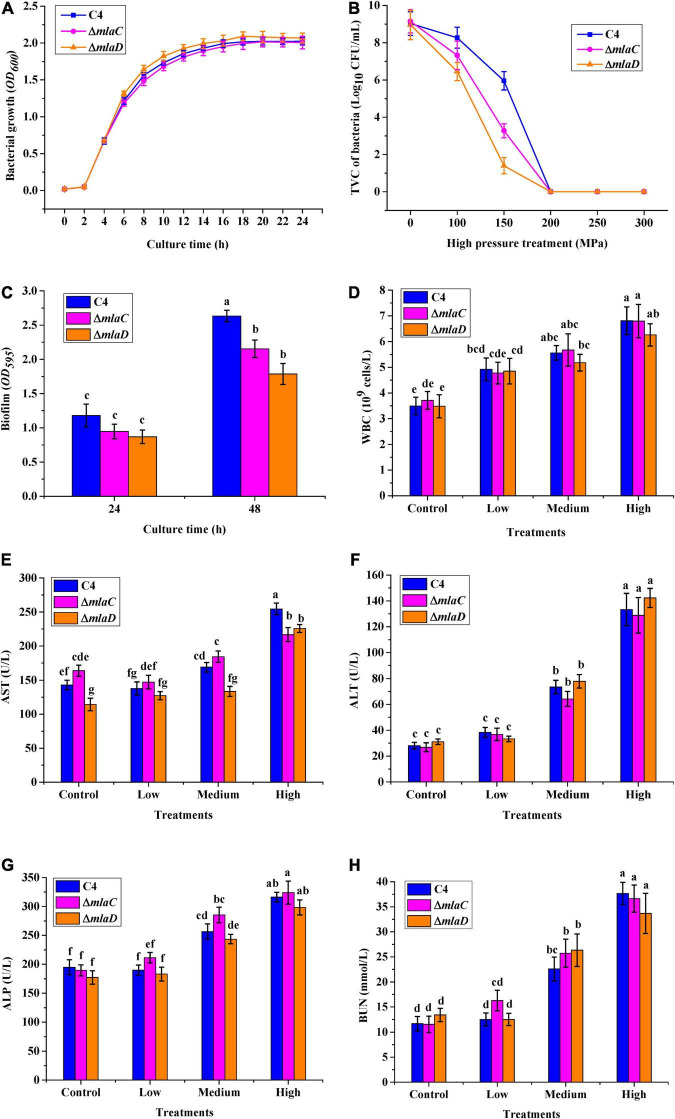
High-pressure tolerance and *in vivo* virulence test of KO strains. **(A)** Growth curve. **(B)** High-pressure tolerance of WT and KO strains under high-pressure treatment. **(C)** Biofilms formed by different strains after culture for 24 and 48 h. **(D)** WBC. **(E)** AST. **(F)** ALT. **(G)** ALP. **(H)** BUN.

To confirm the functions of the *mlaC* and *mlaD* genes, KO strains of Δ*mlaC* and Δ*mlaD* were generated from the WTstrain C4. The growth rates of Δ*mlaC* and Δ*mlaD* were almost the same as that of the C4 strain (Figure 9A). However, these two KO strains had lower tolerance to high pressure. Under the 150 MPa treatment, the cell number of Δ*mlaC* was 1.40 ± 0.43 log_10_ CFU/mL, which was significantly lower than the cell numbers of Δ*mlaD* (3.28 ± 0.37 log_10_ CFU/mL) and WT(5.96 ± 0.50 log_10_ CFU/mL) (*p* < 0.05) (Figure 9B), indicating that the *mlaC* and *mlaD* genes contributed to the high-pressure tolerance of *V. parahaemolyticus*. In addition, the biofilm amounts of the KO strains were significantly lower than that of the C4 strain (Figure 9C), suggesting that the *mlaC* and *mlaD* genes were related to the formation of biofilm, which had an important effect on the tolerance to high-pressure stress.

To verify the relationships between the *mlaC* and *mlaD* genes and bacterial virulence, mice were gavaged with the KO strains, and then five blood parameters, WBC, AST, ALT, ALP, and BUN, were measured. As the density of the three strains increased, the levels of WBC, ALT, ALP, and BUN infection indicators in mice also increased, but there was no significant difference between the WT and KO strains (Figures 9D,F–H). However, compared with the WT strain, the AST levels in mice were significantly lower after infection with medium or high concentrations of the Δ*mlaC* strain, and this level was also lower after infection with a high concentration of the Δ*mlaD* strain (Figure 9E). These results indicated that the *mlaC* and *mlaD* genes were involved in the high-pressure tolerance of cells, and their KO affected the pathogenicity of *V. parahaemolyticus*.

## Discussion

As a Gram-negative halophilic bacterium, *V. parahaemolyticus* is mainly distributed in seafood, such as shellfish, shrimp, and fish, throughout the world (Raszl et al., 2016; Li et al., 2019). In this study, all of the shellfish (oyster, clam, and scallop) samples were contaminated with *V. parahaemolyticus* (Figure 1A), this result might be due to oyster being a bivalve that filter feeds to concentrate microorganisms (Shen et al., 2019). Although *V. parahaemolyticus* can also adhere to the gills and skin of marine fish (e.g., codfish, horse mackerel, and flounder) (Hara-Kudo et al., 2001), it was not detected in the large yellow croaker. Theethakaew et al. (2013) reported that the *tdh* and *trh* genes were present mainly in clinical isolates and rarely in environmental isolates, and Gutierrez West et al. (2013) reported that the proportions of *tdh* and *trh* in environmental samples were 48 and 8.3%, respectively. The C4 strain contained a *tdh* gene, but lacked a *trh* gene, which was consistent with the above reports.

High-pressure processing is a commercialized technology that inactivates or kills microorganisms to extend the shelf life and maintain the good sensory quality of seafood; however, some microorganisms (e.g., *Staphylococcus aureus*, *E. coli*, *Salmonella enterica*) can survive high-pressure treatment (Zhao et al., 2019; Li et al., 2020). In this study, a high-pressure-tolerant strain of N11 was evolved from the wide-type strain of C4 after eight rounds of high-pressure treatment (Figure 2A), and the survival rate of the N11 showed that it could tolerate 200 MPa treatment (Figure 2B). This result was similar with previous reports that adaptive laboratory evolution can be employed as a biological engineering method to obtain cells with tolerance against certain stress conditions (Godara and Kao, 2020); for example, tolerant strains of *Synechocystis* sp. PCC 6803 can grow on medium containing 5 g/L isobutanol (Matsusako et al., 2017), and the acid-adapted *E. coli* K-12 MG1655 strain can maintain a normal growth rate in pH 5.5 medium (Du et al., 2020). Therefore, HPP could lead to the transformation of foodborne pathogenic bacteria into high-pressure-tolerant strain through adaptive laboratory evolution.

To cope with harmful or stressful conditions (e.g., adverse pH, temperature, and osmotic pressure conditions), stress-induced adaptive bacteria undergo physiological and morphological changes (Matsusako et al., 2017; Godara and Kao, 2020). The higher enzyme activities of CAT and Na^+^/K^+^-ATPase helped the N11 strain have strong antioxidant protection and stable ionic and osmotic balance (Mahaseth and Kuzminov, 2017; Kumari and Rathore, 2020). In addition, the cell membrane change of the N11 strain was very obvious. Under high-pressure treatment, the lower membrane zeta potential and intact cell membrane structure effectively reduced the release of cell contents into extracellular space, which improved the survival rate of the N11 strain.

Adaptive laboratory evolution acts on the heritable traits of populations by selecting beneficial alleles and increasing their frequencies while decreasing those of deleterious alleles (Nevado et al., 2019). Some individuals in the population will exhibit genetic mutations that help them survive in unfavorable environmental conditions (Svensson and Berger, 2019). For example, in *E. coli* K-12 MG1655, the *rpoC* gene mutation caused by adaptive laboratory evolution increase its expression level, which in turn endow the bacteria with acid tolerance (Du et al., 2020). In this study, there were 19 mutated nucleotides located in intergenic regions of the N11 strain (Figure 7), the expression levels of the *vp4521* and *vp4522* genes were changed under high-pressure treatment (Figure 8), and the survival rates of Δ*mlaC* and Δ*mlaD* (Figure 9B) confirmed that the *mlaC* and *mlaD* genes were helpful in resisting high-pressure stress. Therefore, such adaptive genetic mutations alter the physiological processes of cells, mainly by optimizing gene expression levels.

The pathogenicity of *V. parahaemolyticus* is mainly dependent on the virulence factors hemolysin, the type III and type VI secretion systems, adhesion factors, lipopolysaccharides, the iron uptake system, proteases, and outer membrane proteins (Li et al., 2019). Although no mutated nucleotides were detected in those virulence genes and their flanking regions in the N11 strain, the pathogenicity of N11 was less than that of the C4 strain according to the mice gavaged with the pathogen (Figure 5), suggesting that some virulence genes might be affected as a consequence of the mutations found in strain N11. KO of the *mlaC* or *mlaD* gene in *V. parahaemolyticus* resulted in lower AST levels in infected mice as compared with mice infected with the WT(Figure 9E), indicating that the intermembrane phospholipid-binding protein MlaC and the outer membrane lipid asymmetry maintenance protein MlaD not only conferred high-pressure tolerance to *V. parahaemolyticus*, but were also associated with the bacteria-induced hepatotoxicity in mouse. This result was similar to those of *Burkholderia cepacia* and *Pseudomonas aeruginosa*, whose Mla pathway genes played important roles in multi-drug and antibiotic tolerance, and mutation in Mla pathway genes lead to reduced virulence in *P. aeruginosa* (Munguia et al., 2017; Bernier et al., 2018).

## Conclusion

A high-pressure-tolerant strain of N11 was acquired from the WT strain of C4 through adaptive laboratory evolution under HPP stress in this study. A possible mechanism of *V. parahaemolyticus* tolerance to HPP was revealed, and the high-pressure-tolerant strain retained pathogenicity, indicating that this pressure-tolerant *V. parahaemolyticus* strain presents health risks during food processing.

## Data Availability Statement

The datasets presented in this study can be found in online repositories. The names of the repository/repositories and accession number(s) can be found in the article/Supplementary Material.

## Ethics Statement

The animal study was reviewed and approved by School Animal Care and Ethics Committee of Zhejiang Gongshang University.

## Author Contributions

LF and HL conceived and designed the research, and wrote the manuscript. LF and MX conducted the experiments. LF, HL, and JZ analyzed the data. All authors have read and agreed to the published version of the manuscript.

## Conflict of Interest

The authors declare that the research was conducted in the absence of any commercial or financial relationships that could be construed as a potential conflict of interest.

## Publisher’s Note

All claims expressed in this article are solely those of the authors and do not necessarily represent those of their affiliated organizations, or those of the publisher, the editors and the reviewers. Any product that may be evaluated in this article, or claim that may be made by its manufacturer, is not guaranteed or endorsed by the publisher.

## Footnotes

1
http://www.ncbi.nlm.nih.gov/sra
