# Supplementary material for: Genetic Basis of High-Pressure Tolerance of a Vibrio parahaemolyticus Mutant and Its Pathogenicity
Source: Front Microbiol. 2022 Mar 31;13:827856. doi: 10.3389/fmicb.2022.827856 (PMC9008460; doi:10.3389/fmicb.2022.827856)
Supplement: Supplementary file 1 [file Data_Sheet_1.docx]

**SUPPLEMENTARY MATERIAL**

**Table S1** IDs and primer sequences of genes for PCR.

**Table S2** Morphological and biochemical characteristics of *V. parahaemolyticus.*

**Table S3** The alignment of *toxR* gene sequences.

**Table S4** General genomic feature of *V. parahaemolyticus* C4 strain.

**Table S5** The COG classification of *V. parahaemolyticus* C4 strain.

**Table S6** Comparison of mutant base pairs in the C4 and N11 strains.

**Figure S1** COG annotation of the genomic sequences of *V. parahaemolyticus* C4 strain.

**Table S1 IDs and primer sequences of genes for PCR**

| Gene ID | primer sequence (5'-3') | Note |
| --- | --- | --- |
| tlh_f | ACTCAACACAAGAAGAGATCGACAA | Identification of *V. parahaemolyticus* |
| tlh_r | GATGAGCGGTTGATGTCCAA |  |
| tdh_f | TCCCTTTTCCTGCCCCC |  |
| trh_r | CGCTGCCATTGTATAGTCTTTATC |  |
| trh_f | TTGCTTTCAGTTTGCTATTGGCT |  |
| trh_r | TGTTTACCGTCATATAGGCGCTT |  |
| 16S_f | GTTTGATCATGGCTCAGATTG |  |
| 16S_r | CTACCTTGTTACGACTTCACC |  |
|  |  |  |
| toxR_f | ACCACTGAAAAGGATAGAT | Amplify *toxR* gene |
| toxR_r | TTTGCTATGGTGCCTA |  |
|  |  |  |
| M13_f | GTTTTCCCAGTCACGAC | Test colony |
| M13_r | CAGGAAACAGCTATGAC |  |
|  |  |  |
| vp2661_f | TGAGAGTAGGACATCGCCAGG | Verify mutant base pairs |
| vp2661_r | CCCAAGAGCCGAGAAACAAC |  |
| vp2828_f | CTTGTTTGCCAGCGAGTAATG |  |
| vp2828_r | AACCGTATCAATCTGTGTGGAC |  |
| vp2943_f | ACCGTGTCTCAGTTCCAGTGT |  |
| vp2943_r | CCCTAACGACGAGCAACTAAG |  |
| vpa1285_f | CTTCAGCAGAGTCGGAGAGGT |  |
| vpa1285_r | CCGTGTCTCAGTTCCAGTGTG |  |
|  |  |  |
| vp2661_q_f | GCGAGGATGTCGGAACCAGTC | qPCR verify expression levels of gene near mutant base pairs, and test KO strains |
| vp2661_q_r | AGCCAAGAACAGGAAAACCACC |  |
| vp2662_q_f | CAGAATTATGGGTTGGCAGC |  |
| vp2662_q_r | GAGTAATGTTACTTACACGACCG |  |
| vp2827_q_f | TTATTTCCAAGTCTGCGATGC |  |
| vp2827_q_r | TTCCCTTCCAAGAAAGACCAT |  |
| vp2828_q_f | CCCTGGACAGATACTGACACTC |  |
| vp2828_q_r | GCCACAACCTCCAAGTAGACAT |  |
| vp2943_q_f | GACACTCAGATGCGAAAGCG |  |
| vp2943_q_r | GCCACAACCTCCAAGTAGACAT |  |
| vp2944_q_f | ACACCATCTATTCAACCAACG |  |
| vp2944_q_r | ATAACGACAAAACCAAACCCT |  |
| vpa1284_q_f | GCCTAAGATAACGTTACTTCTG |  |
| vpa1284_q_r | ATGGGTTTAGAAACCAGCTTTC |  |
| vpa1285_q_f | TCTCTTTCGCTGCTCTGTTTC |  |
| vpa1285_q_r | AGGTAGTTGGTTTCTGGCTCG |  |
| 16s_q_f | GTTGGTGAGGTAAGGGCTCA |  |
| 16s_q_r | GCTGATCATCCTCTCAGACCA |  |
|  |  |  |
| vp2661_up_f1435 | GCAAGGCTATGTAGTGCTGG | KO for *vp2661* |
| vp2661_up_r2495 | AAGCAGCTCCAGCCTACACAAGTTATTCCTTACTTGAACCG |  |
| vp2661_down_f3131 | CTAAGGAGGATATTCATATGCGTAATGGCTCAGTCTCACG |  |
| vp2661_down_r4153 | CACGTAACCATCTTCAAGTGTG |  |
| vp2661_nest_f1445 | GTAGTGCTGGTGGATTACGG |  |
| vp2661_nest_r4146 | CCATCTTCAAGTGTGATGGTTG |  |
|  |  |  |
| vp2662_up_f922 | CCATTTGTGGGACAAGATCC | KO for *vp2662* |
| vp2662_up_r2004 | AAGCAGCTCCAGCCTACACAGATTAATTCCCAAACATCAATG |  |
| vp2662_down_f2494 | CTAAGGAGGATATTCATATGCCTATGTTAGCTAGACTATTAC |  |
| vp2662_down_r3518 | CATGCACTTCTTGAAGGTTTAG |  |
| vp2662_nest_f931 | GGACAAGATCCAATTACCATG |  |
| vp2662_nest_r3509 | CTTGAAGGTTTAGTGCTTCTTC |  |
|  |  |  |
| Cm_f | TGTGTAGGCTGGAGCTGCTT | amplify *Cm* gene cassette |
| Cm_r | CATATGAATATCCTCCTTAG |  |

**Table S2 Morphological and biochemical characteristics of *V. parahaemolyticus***

| **Reaction** | **Result** | **Reaction** | **Result** | **Reaction** | **Result** |
| --- | --- | --- | --- | --- | --- |
| Arabinose | + | H_2_S | - | ONPG | - |
| Arginine dihydrolase | - | Indole | + | Ornithine decarboxylase | + |
| gelatin | + | Inositol | - | Oxidase | + |
| Glucose | + | Lactose | - | Rhamnose | - |
| Growth in 0% NaCl | - | Lysine decarboxylase | + | Simmons citrate | + |
| Growth in 3% NaCl | + | Methyl red | + | Sucrose | - |
| Growth in 6% NaCl | + | Maltose | + | Voges-Proskauer | - |
| Growth in 8% NaCl | + | Mannitol | + | 42 °C | - |
| Growth in 10% NaCl | - | Mannose | + |  |  |

**Table S3 The alignment of *toxR* gene sequences**

|  | Identity | | | | | | | |
| --- | --- | --- | --- | --- | --- | --- | --- | --- |
|  | C4 | *V. parahaemolyticus* RIMD 2210633 | *V. parahaemolyticus* U-5474 | *V. parahaemolyticus* AQ3810 | *V. parahaemolyticus* vphy216 | *V. campbellii* BAA-1116 | *V. alginolyticus* NBRC 15630 | *V. natriegens* 534 |
| C4 | 100 |  |  |  |  |  |  |  |
| *V. parahaemolyticus* RIMD 2210633 | 99 | 100 |  |  |  |  |  |  |
| *V. parahaemolyticus* U-5474 | 99 | 99 | 100 |  |  |  |  |  |
| *V. parahaemolyticus* AQ3810 | 99 | 99 | 100 | 100 |  |  |  |  |
| *V. parahaemolyticus* vphy216 | 99 | 100 | 99 | 99 | 100 |  |  |  |
| *V. campbellii* BAA-1116 | 84 | 0 | 0 | 0 | 0 | 100 |  |  |
| *V. alginolyticus* NBRC 15630 | 90 | 92 | 92 | 92 | 92 | 0 | 100 |  |
| *V. natriegens* 534 | 0 | 0 | 0 | 0 | 0 | 0 | 0 | 100 |

**Table S4 General genomic feature of** ***V. parahaemolyticus* C4 strain**

| Feature | No. |
| --- | --- |
| Genome Size (bp) | 5,271,691 |
| Scaffolds | 12 |
| (G+C)/(A+G+C+T) | 45.3 |
| scaffold N50 (bp) | 616,582 |
| contig N50 (bp) | 348,586 |
| ORF | 4,850 |
| tRNA | 110 |
| rRNA | 11 |

**Table S5 The COG classification of *V. parahaemolyticus* C4 strain**

| Function class | Description | Gene No. |
| --- | --- | --- |
| A | RNA processing and modification | 1 |
| B | Chromatin structure and dynamics | 4 |
| C | Energy production and conversion | 320 |
| D | Cell cycle control, cell division, chromosome partitioning | 94 |
| E | Amino acid transport and metabolism | 566 |
| F | Nucleotide transport and metabolism | 126 |
| G | Carbohydrate transport and metabolism | 359 |
| H | Coenzyme transport and metabolism | 265 |
| I | Lipid transport and metabolism | 143 |
| J | Translation, ribosomal structure and biogenesis | 235 |
| K | Transcription | 433 |
| L | Replication, recombination and repair | 184 |
| M | Cell wall/membrane/envelope biogenesis | 310 |
| N | Cell motility | 203 |
| O | Posttranslational modification, protein turnover, chaperones | 250 |
| P | Inorganic ion transport and metabolism | 385 |
| Q | Secondary metabolites biosynthesis, transport and catabolism | 190 |
| R | General function prediction only | 742 |
| S | Function unknown | 384 |
| T | Signal transduction mechanisms | 356 |
| U | Intracellular trafficking, secretion, and vesicular transport | 152 |
| V | Defense mechanisms | 173 |
| W | Extracellular structures | 0 |
| Y | Nuclear structure | 0 |
| Z | Cytoskeleton | 0 |

**Table S6 Comparison of mutant base pairs in the C4 and N11 strains**

| **Region** | **Strain** | **Base pairs** | | | | | | |
| --- | --- | --- | --- | --- | --- | --- | --- | --- |
| Between *vp2661* and *vp2662* | C4 | A | G | A | G |  |  |  |
|  | N11 | G | A | T | A |  |  |  |
| Between *vp2827* and *vp2828* | C4 | G |  |  |  |  |  |  |
|  | N11 | A |  |  |  |  |  |  |
| Between *vp2943* and *vp2944* | C4 | C | T | C | G | A | A | A |
|  | N11 | T | C | T | T | T | C | G |
| Between *vpa1284* and *vpa1285* | C4 | C | C | G | C | T | G | A |
|  | N11 | A | A | T | T | C | A | T |


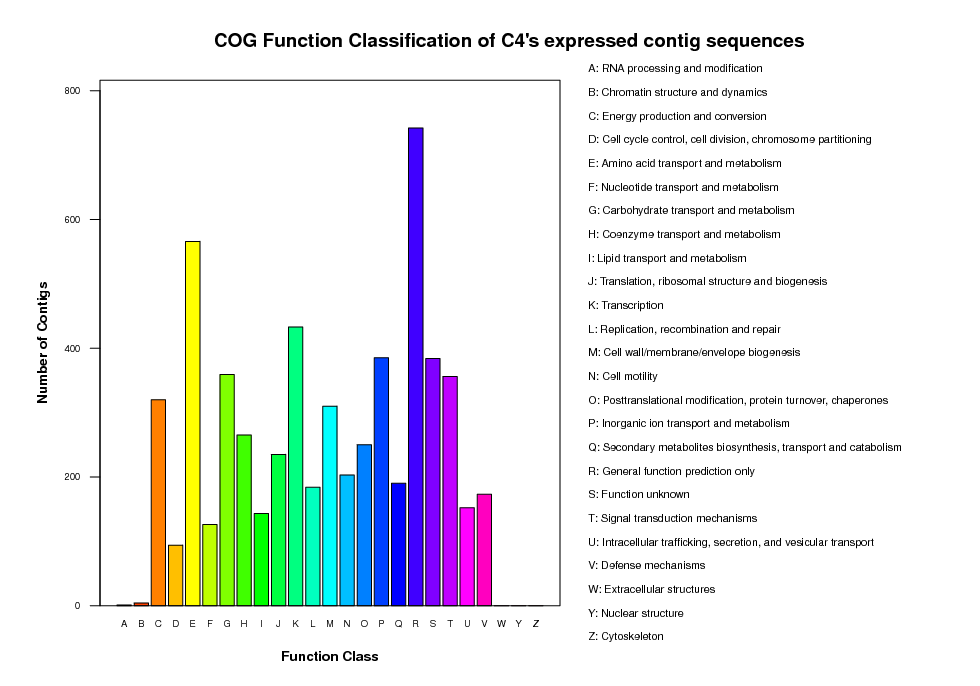


**Figure S1** COG annotation of the genomic sequences of *V. parahaemolyticus* C4 strain
